# Iatrogenic Complete Anterolateral Papillary Muscle Rupture Following Transcatheter Gelatin Sponge Embolization for Coronary Artery Perforation

**DOI:** 10.1016/j.jaccas.2025.106610

**Published:** 2026-01-21

**Authors:** Hiroki Yokomori, Hiroki Niikura, Yuki Yokouchi, Shiho Ide, Go Hashimoto, Raisuke Iijima, Kei Takahashi, Masato Nakamura, Hidehiko Hara

**Affiliations:** a Division of Cardiology, Toho University Ohashi Medical Center, Tokyo, Japan; b Division of Pathology, Toho University Ohashi Medical Center, Tokyo, Japan; c Division of Minimally Invasive Treatment in Cardiovascular Medicine, Toho University Ohashi Medical Center, Tokyo, Japan

**Keywords:** acute mitral regurgitation, guidewire perforation, papillary muscle rupture, percutaneous coronary intervention, transcatheter embolization

## Abstract

**Background:**

Iatrogenic papillary muscle rupture (iPMR) is a rare but fatal complication of catheter interventions. No previous report to our knowledge has described iPMR with pathologic findings following transcatheter embolization for guidewire perforation of coronary artery.

**Case Summary:**

An 83-year-old man with unstable angina underwent a percutaneous coronary intervention. On the same night as the percutaneous coronary intervention, a guidewire perforation of the left circumflex artery side branch was identified, resulting in cardiac tamponade. Transcatheter embolization of the culprit vessel was performed using gelatin sponge particles. Severe acute mitral regurgitation with circulatory collapse developed 30 hours later secondary to anterolateral iPMR. Despite mechanical circulatory support, the patient died 2 days after mitral regurgitation onset.

**Discussion:**

Pathologic findings revealed embolic materials within the anterolateral papillary muscle, causing new papillary muscle necrosis and rupture.

**Take-Home Message:**

Gelatin sponge embolization may achieve hemostasis in coronary guidewire perforation but poses a significant risk of iPMR.

## Visual Summary

**Figure:**
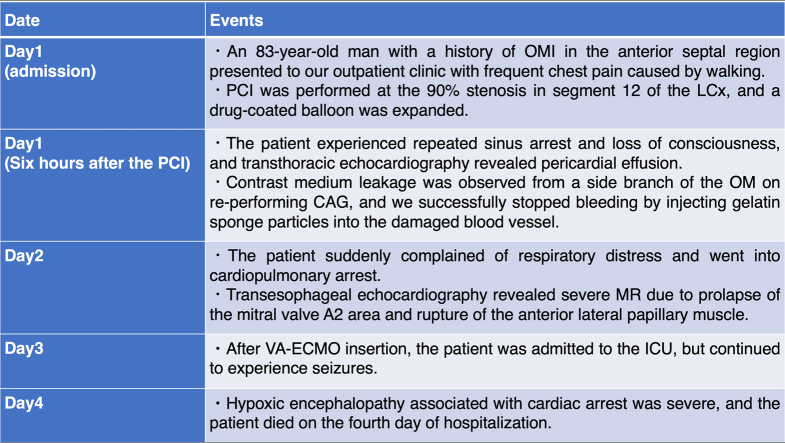

## History of Presentation

An 83-year-old man presented at our outpatient clinic with frequent chest pain. Initial evaluation revealed a normal body temperature, peripheral oxygen saturation of 97% (room air), blood pressure of 122/42 mm Hg, and a heart rate of 54 beats/min. However, no murmur was detected. Mild edema was observed in the lower legs. Electrocardiography revealed a sinus rhythm with no ST-segment changes. Chest radiography revealed a cardiothoracic ratio of 52% without pulmonary congestion or pleural effusion. His creatine kinase-MB (CK-MB) level was 3.3 ng/mL, and high-sensitivity troponin T level was 0.037 ng/mL (upper normal limit: 0.014). Transthoracic echocardiography revealed a decreased left ventricular ejection fraction of 45% and localized wall motion abnormalities in the anterior septal region. No significant valvular disease or pericardial effusion was observed.

**Take-Home Messages**

• Transcatheter hemostatic embolization for guidewire coronary perforations using gelatin sponge hemostatic particles can be effective for rapid hemodynamic stabilization; however, it may lead to a serious risk of iatrogenic ischemia and rupture of the papillary muscle.
• All invasive procedures can cause fatal complications; therefore, careful consideration must be given before deciding to perform these procedures.

## Past Medical History

The patient had a history of old myocardial infarction (MI) in the anterior septal region and had undergone percutaneous coronary intervention (PCI) for segment 6 of the left anterior descending coronary artery (LAD) with placement of a drug-eluting stent 7 years previously. The patient also had a history of hypertension, dyslipidemia, and chronic kidney disease.

**Visual Summary:**
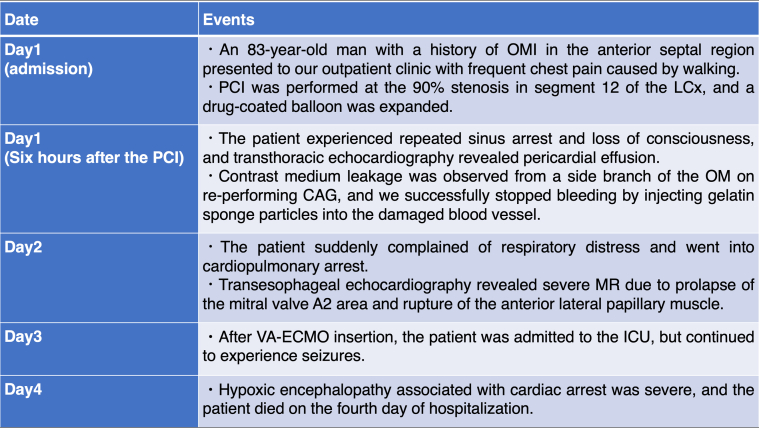
Timeline of Case Presentation CAG = coronary angiography; LCx = left circumflex artery; MR = mitral regurgitation; PCI = percutaneous coronary intervention; VA-ECMO = venoarterial extracorporeal membrane oxygenation.

## Differential Diagnosis

The patient's chest pain gradually worsened and became more frequent with each passing day, and the high-sensitivity troponin T level was slightly elevated. We diagnosed unstable angina and decided to perform emergency coronary angiography (CAG) on the same day.

## Investigations

**Figure 1:**
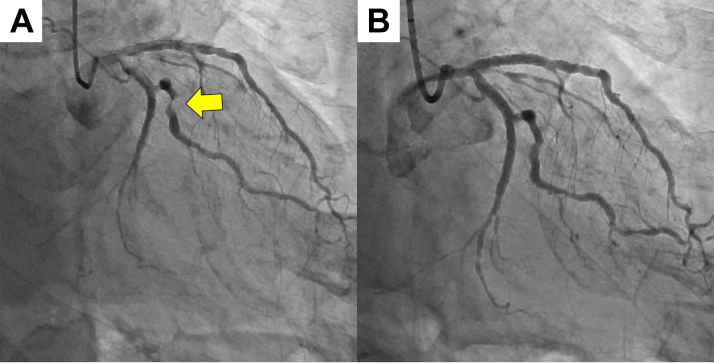
PCI Using a Drug-Coated Balloon on Unstable Angina Pectoris (A) Coronary angiography was performed on admission. The culprit lesion was the left circumflex artery (arrow). (B) Coronary angiography after percutaneous coronary intervention (PCI).

**Figure 2:**
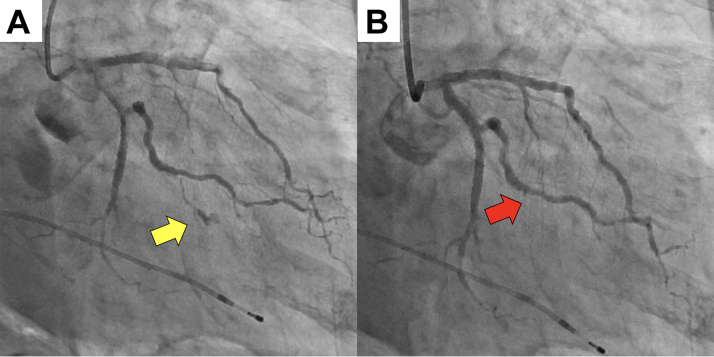
Coronary Artery Embolization Using Gelatin Sponge Hemostatic Particles for Treating Guidewire Perforation (A) Repeat coronary angiography on the night of admission. Contrast medium leakage was observed in a side branch of the obtuse ramus (yellow arrow). (B) Coronary angiography after achieving hemostasis. The damaged vessel was occluded by the injection of gelatin sponge hemostatic particles (red arrow).

Emergency CAG revealed 90% stenosis in segment 12 of the left circumflex artery (LCx) (Figure 1A). PCI was performed at this site using a 2.5-mm drug-coated balloon (Figure 1B). Six hours after PCI, despite postoperative blood tests showing no elevation in CK-MB levels, the patient experienced repeated sinus arrest and loss of consciousness. A temporary pacemaker was inserted into the right side of the neck. Transthoracic echocardiography revealed a new pericardial effusion. On repeat CAG, contrast leakage was observed from a small side branch of the obtuse ramus (Figure 2A). We identified that a coronary artery perforation, resulting from an injury caused by the coronary guidewire, occurred during the most recent PCI procedure. We attempted to stop the hemorrhage by inserting a microcatheter into the injured small branch vessel and applying negative pressure. However, because this procedure was unsuccessful and contrast extravasation persisted, we performed embolization of the injured small vessel using gelatin sponge hemostatic particles. The particles were prepared by manually cutting a sterile hemostatic gelatin sponge into pieces of <1 mm in diameter and stirring them in a mixture of saline and contrast. The microcatheter was advanced deep into the target small vessel to prevent backflow of embolization particles into the main LCx branch. Gelatin sponge particles were injected into the target vessels, followed by a flush with normal saline (1.5 mL). After the first injection, the target vessel was completely occluded, and hemostasis was achieved (Figure 2B). Angiography confirmed that embolization had not occurred in any vessel other than the target vessel. Pericardial drainage was performed owing to persistent hypotension, and 350 mL of pericardial fluid was obtained. The following day, CK-MB was 138 U/L. There was almost no effusion from the pericardial drain, and hemodynamics were stable. However, approximately 30 hours after transcatheter embolization, the patient suddenly reported respiratory distress and experienced cardiopulmonary arrest.

## Management

**Figure 3:**
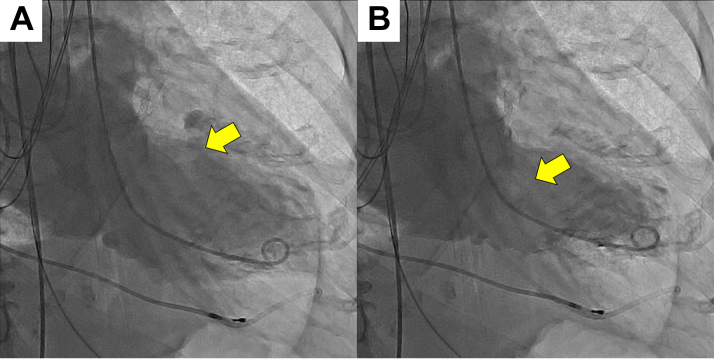
Left Ventriculography Was Performed at Time of Acute Deterioration (A) The left atrium was opacified more intensely than the left ventricle, corresponding to Sellers classification grade 4. (B) A ruptured anterolateral papillary muscle was observed moving within the left ventricular cavity (yellow arrow).

**Figure 4:**
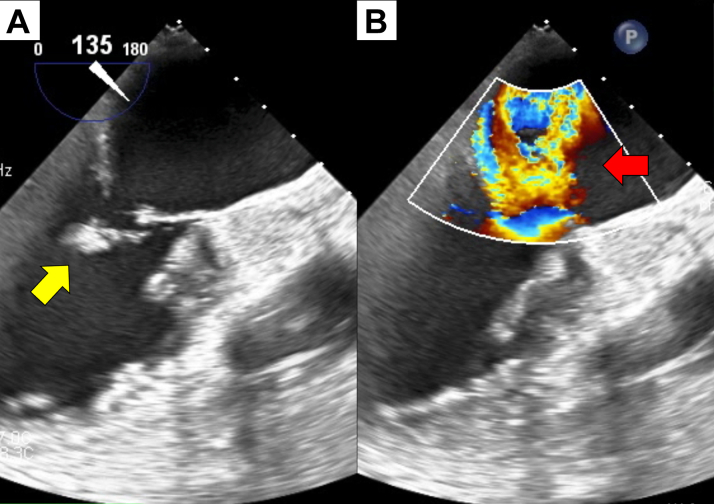
Severe Mitral Regurgitation Due to Papillary Muscle Rupture (A) Transesophageal echocardiography demonstrating the presence of papillary muscle rupture (yellow arrow). (B) Transesophageal echocardiography demonstrating the presence of severe mitral regurgitation (red arrow).

Cardiopulmonary resuscitation was immediately initiated; however, hemodynamics remained extremely unstable, and cardiac arrest recurred, prompting venoarterial extracorporeal membrane oxygenation. Repeat CAG revealed no coronary lesions or contrast leakage from the previously injured vessels. Left ventricular angiography revealed papillary muscle rupture (PMR) and mitral regurgitation (MR), which was grade 4 according to the Sellers classification (Figure 3). Acute heart failure secondary to acute severe MR was diagnosed. Transesophageal echocardiography revealed severe MR due to complete rupture of the anterior lateral papillary muscle (ALPM) (Figure 4).

## Follow-Up

**Figure 5:**
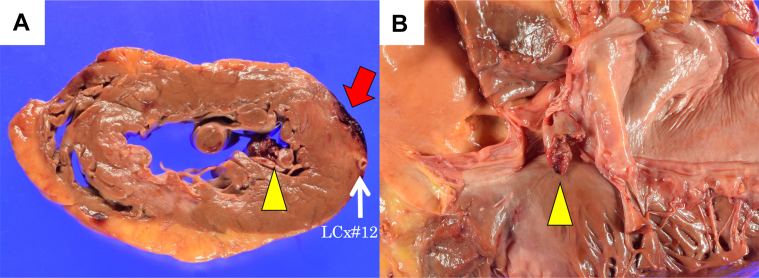
ALPM Rupture on Macroscopic Pathologic Examination (A) The heart was horizontally sectioned at the level of the papillary muscles. Bleeding was observed in the side branch area of the obtuse ramus (red arrow). The anterior lateral papillary muscle (ALPM) was also ruptured (yellow arrowhead). (B) The heart was opened. Rupture of the ALPM was observed (yellow arrowhead).

**Figure 6:**
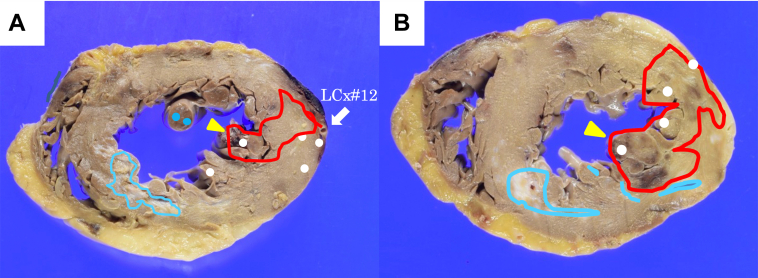
Area and Location of MI and Gelatin Sponge Hemostatic Particles Areas affected by acute myocardial infarction (MI) (red line) and old MI (light blue line) and the area where the gelatin sponge hemostatic particles were confirmed inside the blood vessel (white dot). (A) Papillary muscle level. (B) At the level of the left ventricular free wall.

**Figure 7:**
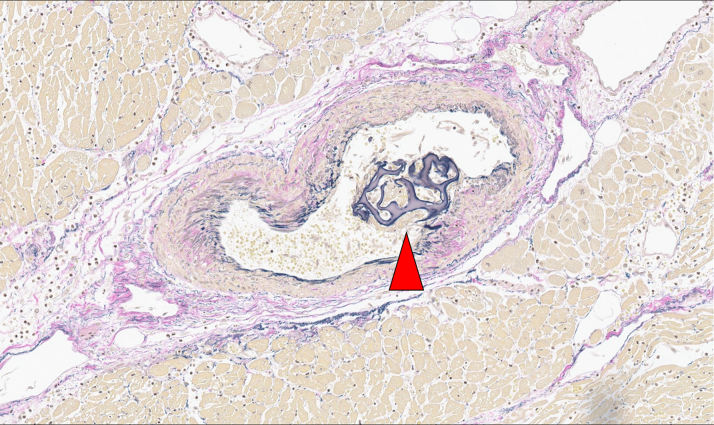
Prevalence of Gelatin Sponge Hemostatic Particles in the Nutrient Artery of the ALPM Pathologic microlevel demonstrated gelatin sponge hemostatic particles, evident in the nutrient artery of the anterior lateral papillary muscle (ALPM) (red arrow).

After initiation of venoarterial extracorporeal membrane oxygenation, the patient was admitted to the intensive care unit but continued to experience seizures resulting from severe hypoxic encephalopathy after cardiac arrest. We explained and discussed the situation with the family and heart team and decided against aggressive treatment. The patient died 2 days after the onset of severe acute MR. A subsequent pathologic autopsy revealed the following findings: Gross examination revealed a complete rupture of the ALPM (Figure 5). The ALPM also showed only an acute infarct area, without evidence of prior infarction (Figure 6). In addition, gelatin sponge hemostatic particles were identified within the ALPM supply vessel (Figure 7). These findings suggest that transcatheter embolization with hemostatic gelatin sponge particles can cause iatrogenic PMR.

## Discussion

We report a case in which transcatheter embolization using gelatin sponge hemostatic particles was used to control guidewire perforation of coronary artery. This embolization caused complete iatrogenic ALPM rupture, leading to acute severe MR with circulatory collapse. To our knowledge, this is the first case report of pathologically confirmed iatrogenic PMR secondary to coronary embolization using gelatin sponge particles.

Complications occur in a significant proportion of PCI procedures. Among these, guidewire perforation is uncommon (incidence <1%), but it carries a high in-hospital mortality of approximately 4% to 8% when coronary perforation occurs.^1^, ^2^, ^3^ Consequently, conservative observations were discouraged. The standard first-line strategy is to attempt hemostasis by flow occlusion, such as maintaining continuous negative pressure using a microcatheter, prolonging inflation with a perfusion balloon, or deploying a covered stent. If the hemorrhage remains uncontrolled or the risk of rebleeding is high after the flow-occlusion strategy, the next recommended approach is embolization of the perforation site with coils, fat tissue, or other embolic materials to stabilize hemodynamics.^4^^,^^5^ In the present case, conventional strategies failed to achieve hemostasis, necessitating transcatheter embolization using embolic materials. Coils are often used as embolization material; however, we had no choice but to use gelatin sponge hemostatic particles as a last resort because we did not have appropriate coils available for the culprit small vessel with bleeding. Gelatin sponges provide immediate hemostasis by mechanically packing the vessel lumen, thereby promoting clot formation. However, its use poses a significant risk of permanent vessel occlusion and iatrogenic MI, regardless of particle size, depending on particle amount and compactness.^6^ In our patient, despite selective minimum embolization, complete iatrogenic ALPM rupture occurred because of an iatrogenic MI.

Although PMR causing acute MR is rare, its onset is typically catastrophic, with abrupt hemodynamic decompensation and acute heart failure. Iatrogenic PMR has previously been reported after procedures such as transcatheter aortic valve replacement and percutaneous transseptal mitral commissurotomy,^7^^,^^8^ but not after embolization for coronary perforation using gelatin sponge hemostatic particles. In addition, the ALPM is generally supplied by 2 branches (ie, LAD and LCx), making ALPM ruptures rare.^9^ However, in this case, the patient had a prior anterior wall MI in the LAD, yet the ALPM showed no fibrosis, indicating that the previous MI did not damage the ALPM. Pathologic findings demonstrated fresh myocardial necrosis tracking the course of an LCx branch and embolic materials within small intramyocardial vessels supplying the ALPM, establishing a direct link between hemostatic embolization and iatrogenic PMR with acute MR. Additionally, embolic materials were identified proximal to the perforated segment, implying excessive embolization and suggesting that the iatrogenic infarct size may have been amplified by dispersion into vessels beyond the intended branch. These findings raise the possibility that an excessive amount of gelatin sponge particles were delivered.

For PMR with hemodynamic collapse, the standard treatment is prompt mechanical circulatory support followed by surgical or transcatheter mitral valve repair or replacement because acute MR from PMR has a poor prognosis.^10^ In our case, the delayed return of spontaneous circulation and evidence of severe hypoxic-ischemic encephalopathy led the family to decline mitral intervention.

This pathologically confirmed case highlights that even when hemostasis for guidewire perforation is achieved, hemodynamic deterioration may arise from mechanisms other than cardiac tamponade. Therefore, vigilant postprocedural monitoring should be performed for both tamponade and nontamponade causes of shock. Additionally, interventionalists must recognize the risk of excessive embolization of gelatin sponge hemostatic particles into the coronary arteries, as this can result in iatrogenic ischemia and rupture of the papillary muscles. To reduce this risk, interventionalists should optimize gelatin sponge particle dosing and ensure precise distal-target delivery, as confirmed angiographically. In addition, this procedure must be performed with the understanding that it causes iatrogenic infarction around the target vessel, and it is important to carefully observe not only hemostasis but also coronary artery perfusion, such as surrounding blood flow and delayed blush.

## Conclusions

Transcatheter embolization using gelatin sponge hemostatic particles for coronary perforation may be effective; however, interventionalists should recognize the potential for severe postprocedural complications, including iatrogenic PMR.

## Funding Support and Author Disclosures

The authors have reported that they have no relationships relevant to the contents of this paper to disclose.
